# The role of contextual factors, school-related attitudes and behavioural traits on lifetime antisocial behaviours among adolescents

**DOI:** 10.3389/fpubh.2026.1903347

**Published:** 2026-09-29

**Authors:** Emina Mehanović, Mariaelisa Renna, Marco Martorana, Erica Viola, Alberto Sciutto, Serena Vadrucci, Maria Ginechesi, Claudia Vullo, Adalgisa Ceccano, Pietro Casella, Fabrizio Faggiano, Federica Vigna-Taglianti

**Affiliations:** 1 Department of Sustainable Development and Ecological Transition, University of Piemonte Orientale, Vercelli, Italy; 2 Department of Translational Medicine, University of Piemonte Orientale, Novara, Italy; 3 Department of Statistics, Computer Science, and Applications “Giuseppe Parenti” (DiSIA), University of Florence, Florence, Italy; 4 Department of Psychology, eCampus University, Novedrate, Italy; 5 Department of Prevention, Hygiene and Public Health Unit, ASL Città di Torino, Torino, Italy; 6 Department of Mental Health, Addiction Unit, ASL Roma1, Roma, Italy; 7 Epidemiology Unit, ASL Vercelli, Vercelli, Italy

**Keywords:** adolescents, antisocial behaviour, impulsiveness, parental permissiveness, parental support, peer’s substance use, school factors, sensation-seeking

## Abstract

**Introduction:**

Antisocial behaviour is a result of a multifactorial process that involves both social-level and individual-level factors. The present study aims to investigate the association of contextual factors with the odds of engaging in antisocial behaviours, and to explore the role of school-related attitudes and behavioural traits in the association between contextual factors and antisocial behaviour among 12- to 14-year-old adolescents.

**Methods:**

The analytical sample included 1847 students of 29 secondary schools in Italy who participated in the baseline survey of the “GAPUnplugged” study. The associations of sociodemographic characteristics, contextual factors, school-related attitudes and behavioural traits with the odds of adolescent’s antisocial behaviour were estimated through multilevel mixed-effect models. Exploratory indirect association models were performed to test the potential mediation role of school-related attitudes and behavioural traits in the relationship between contextual factors and adolescent’s antisocial behaviour.

**Results:**

The prevalence of at least one antisocial behaviour lifetime was 61.2%, of two 39% and of three or more antisocial behaviours 21.8%. Among contextual factors, perceived parental permissiveness to use substances, low perceived parental support, low perceived class climate, and perceptions of peer’s cigarette, alcohol and illicit drug use were associated with higher odds of antisocial behaviour. Low pupil’s respect for teacher, high impulsiveness and sensation-seeking scores were significant indirect correlates of most contextual factors on antisocial behaviours.

**Conclusion:**

Contextual factors, school-related attitudes and behavioural traits have a strong influence on antisocial behaviours in adolescence. All these factors should be considered in prevention interventions aimed at reducing or preventing antisocial behaviour among early adolescents.

## 1 Introduction

Antisocial behaviours are defined as aggressive and rule-breaking behaviours, harming the rights of other people and violating the societal norms. Violence, bullying, stealing and vandalism acts are examples of antisocial behaviours studied in the literature (1). They co-occur commonly with other risk behaviours such as alcohol intoxication, cannabis use and other illicit drug use, but also with mental health disorders such as ADHD and depression (2–7). Antisocial behaviours do not only increase the risk of negative consequences for adolescent himself/herself, but also pose a distress on others, often requiring the involvement of authorities and criminal justice system. The ESPAD 2019 survey reported that 18.3% of 15–17 years-old Italian adolescents engaged in violent behaviours (3).

Antisocial behaviour has been described in the literature as a result of a multifactorial process, with both social-level and individual-level factors involved in the development of such behaviours in adolescents (8). Social-level factors include socioeconomic conditions, family climate and parental behaviours, peer norms, school factors, and other aspects of adolescent’s environment that can influence the adoption of risk and protective behaviours. Among parental factors, previous studies identified low socioeconomic status, low parental monitoring, low parental support, absence of parents, weak attachment to parents, family conflicts, poor family management, unhealthy family functioning, parental permissive style, disruptive families, negative family climate, family history of substance use and antisocial behaviours, parental attitudes favourable to drug use and antisocial behaviour, and parental mental health problems as factors associated with the odds of adopting antisocial behaviours in adolescents (3, 4, 6, 7, 9–15). Among peer factors, affiliation with antisocial peers, and peer’s alcohol and drug use were strongly related with an increased likelihood of engaging in antisocial behaviour (4, 5, 9, 11, 12, 16).

Studies on trajectories showed that social-level factors can influence adolescent’s engagement in antisocial behaviours through some individual-level factors, i.e., personality traits and factors directly related to one’s individual’s daily experiences (15). Among these, sensation-seeking attitude and impulsivity, low empathy, anger reactivity and low self-efficacy were found to increase the susceptibility to rule-breaking behaviours (6, 9, 11–13, 16, 17). Also weak school bonding, academic failure and low commitment to school were found to be associated with antisocial behaviour in adolescents (4, 9, 11, 12). By definition, all these factors that are directly associated with antisocial behaviour can have a role in the trajectory between social-level factors and the actual behaviour. A study conducted in US found that intention to attend college, desire to leave home, use of alcohol and drugs, group fighting, and baseline violence mediated the effect of parent- and peer-level variables on violent adolescent behaviour (15). The study by Houltberg et al. (13) found that anger reactivity mediated the relationship between parental permissive style and antisocial behaviour in a sample of adolescents residing in disadvantaged neighbourhoods. In the study by Piotrowska et al. (14), unhealthy family functioning, stressful life events, literacy difficulties and neighbourhood disadvantage mediated the effect of income on antisocial behaviours. To extend previous findings, the present study aims to investigate the association of contextual factors (parental factors, class climate, and peer’s norms) as perceived by the adolescent with the probability of involvement in antisocial behaviours. The second aim is to investigate the role of school-related attitudes and behavioural traits in the relationship between contextual factors and antisocial behaviour in a sample of 12- to 14-year-old Italian adolescents.

## 2 Materials and methods

### 2.1 Study design and sample

This study used data from the baseline survey of the experimental controlled trial “GAPUnplugged” designed to evaluate the effectiveness of the school prevention curriculum “Unplugged” integrated with new gambling content for preventing gambling behaviours among 12- to 14-year-s old Italian adolescents. Study design and methods of the trial are described elsewhere (18). The baseline survey involved 1874 students of 29 secondary schools in 9 NHS districts of Piedmont Region (Alessandria, Torino 3, Torino 5, Vercelli, Cuneo 1, Cuneo 2, city of Torino, and Novara) and Rome city between November 2022 and January 2023. The analytical sample included 1847 students who provided the answer to the question of antisocial behaviour in lifetime.

### 2.2 Data collection

A self-completed anonymous questionnaire was used to collect information on sociodemographic characteristics, substance use, gambling behaviours, personal factors and skills, sensation-seeking, impulsiveness, the perception of peers’ and friends’ substance use and gambling, friend’s approval of gambling, parental gambling, parental factors, parental permissiveness toward substance use and gambling, class climate, relation with mathematics, grades, and antisocial behaviours. All the information was self-reported by the students; therefore, all the variables are a reflection of adolescent’s subjective perception. Only students whose parents or caregivers gave consent to participate were involved in the study. Before the administration of questionnaires, information on the study was provided to the pupils and consent to participate was asked. The questionnaire was developed *ad hoc* and included previously validated questions derived from the EU-Dap survey^1^, the EDDRA data bank of EUDA^2^, and other international sources and projects (ESPAD, HBSC, Project ALERT, RATING Swedish cohort, SOGS-RA Italian validated version, BSSS Italian version). To preserve confidentiality of the data a 9-digit individual code was self-generated by the student. The questionnaires were filled in by students in the classroom during the school time through an online application. In cases of lack of computers or problems of connection, the researchers administered the paper version of the questionnaire.

### 2.3 Measures

Individual socio-demographic information included gender, age (based on birth date), family composition (living with “both parents,” “one parent,” and “others”) and languages spoken in family (“Italian” and “Others”). Mother and father occupation was assessed by asking “What job does your mother do?” and “What job does your father do?”. Based on the answers the student provided we created an indicator of socioeconomic status defined “Household employment status”, categorised as “Two salaries”, “One salary” and “No salary.”

Antisocial behaviour was investigated by asking students if they ever “Hit one of your teachers”, “Got into fight with someone”, “Seriously hurt someone”, “Threat with a weapon of any kind to get something from someone”, “Destroyed on purpose someone’s items”, “Stole something” and “Sold stolen items”, with response categories “Yes” and “No.” Since the questionnaire assessed lifetime occurrence of each behaviour without capturing severity or number of times, antisocial behaviour was operationalised as a dichotomous indicator (yes vs. no), reflecting any involvement in antisocial behaviours. A confirmatory factor analysis confirmed the unidimensionality of the scale. The reliability of the score, based on tetrachoric correlations, was good (ordinal *α* = 0.831). Composite reliability suggested acceptable reliability (omega = 0.678).

Perceived parental permissiveness was assessed by asking students if their parents would allow them smoking or drinking alcohol (separate questions), with possible responses “Would not allow at all”, “Would not allow at home”, “Would allow.” The two questions were merged into one variable “Parental permissiveness to use substances” with two levels: “Would not allow at all” and “Would not allow at home/Would allow”, the last including the perception of parental permissiveness towards at least one substance.

Perceived parental support was investigated through the single-item statement “I can easily get support from my father and/or mother”, allowing the response alternatives on a 4-point Likert scale (strongly agree/agree/disagree/strongly disagree). The answers were summed up into a dichotomous indicator “High” and “Low.”

Perceived class climate was assessed through the statements “The students in my class enjoy being together,” “Most of the students in my class are kind and helpful” and “Other students accept me as I am”. The reliability of the score was good (Cronbach’s α = 0.728).

Perception of peer’s cigarette, alcohol and illicit drug use (three separate questions) allowed the answers “None,” “Less than half of them,” “About half of them”, “More than half of them” and “All of them”. Answers were collapsed into a dichotomous variable “Yes” and “No” for each indicator.

School motivation was assessed by a statement “How I do in school matters a lot to me,” whereas pupil’s respect for teachers was assessed by a statement “I have great respect for what my teachers tell me,” allowing the response alternatives on a 4-point Likert scale (strongly agree/agree/disagree/strongly disagree). The answers were summed up into a dichotomous indicator “High” and “Low.”

Impulsivity was measured though five items retrieved from the Eyseneck Impulsiveness scale (19), later on used by Vitaro et al. (20): “I often say or do things without thinking,” “I often get in troubles because I do things without thinking it through,” “I am impulsive person,” “I weight up all the choices before I decide on something,” and “I often say something off the top of my head.” The reliability of the score was good (Cronbach’s α = 0.770).

Sensation-seeking was evaluated with the Italian version of Brief Sensation Seeking Scale (BSSS), including questions on experience seeking (e.g., “I would like to explore strange places”), boredom susceptibility (e.g., “I get restless when I spend too much time at home”), thrill and adventure seeking (e.g., “I like to do frightening things”), and disinhibition (e.g., “I like wild parties”) (21). The reliability of the scale was good (Cronbach’s α = 0.760).

Cigarette smoking was measured by asking students if they smoked cigarettes during lifetime, with possible answers “Yes” and “No”. Alcohol drinking was measured by asking students if they drank alcohol during lifetime, with possible answers “Yes” and “No.” Gambling behaviour was investigated by asking students if they gambled (scratch cards, lottery, bingo, slot machines, sport betting, event betting, poker, and cards) during the last 12 months, with response categories ranging on a scale from 0 to 13 times or more for each specific game. A unique variable of any gambling behaviour was created, and all the answers were summed up into a dichotomous indicator “Yes” and “No.”

Questions on perceived class climate, impulsiveness and sensation-seeking allowed response alternatives on a 4-point Likert scale (very likely/likely/unlikely/very unlikely and strongly agree/agree/disagree/strongly disagree). The answers were scored 1–4 and summed, means were calculated, and categories of high, middle, and low level of each indicator were created by using tertiles.

All contextual factors (perceived parental permissiveness to use substances, perceived parental support, perceived class climate, perception of peer’s cigarette, alcohol and illicit drug use) were measured through adolescents’ perceptions of their parents, peers, and school environment.

### 2.4 Statistical analysis

The outcome under study was antisocial behaviour in lifetime (yes/no).

Sociodemographic characteristics, contextual factors (perceived parental permissiveness to use substances, perceived parental support, perceived class climate, perception of peer’s cigarette, alcohol and illicit drug use), school-related attitudes (school motivation and respect for teacher) and behavioural traits (impulsiveness and sensation-seeking), and co-occurrent substance use and gambling behaviours were described through descriptive analysis and summarised as frequency and percentage for categorical variables, and as mean and SD for continuous variables.

The associations of contextual factors with the probability of adolescent’s antisocial behaviour in the lifetime were estimated through multivariate regression models adjusting for confounders (gender, age, household employment status, family structure, languages in family, alcohol drinking, cigarette smoking and gambling). Collinearity between variables was checked before building the final models. Since the perceptions of peer’ cigarette, alcohol and illicit drugs use were collinear, three multivariable models were built, each including one of the collinear variables. In all models, the associations were estimated adjusting for the same confounders. Variables significantly related with the outcome according to bivariate model and non-collinearity were included in the final multivariate regression model simultaneously. Missing data were less than 2% for all studied variables, except for gender (5.6%), perceptions of peer’s alcohol (2.2%) and illicit drug (2.8%) use. Applying listwise deletion to handle missing data, the final multilevel models were run on 88–90% of the initial sample.

Multilevel mixed-effect modelling was used to control for the hierarchical nature of the data, with students nested within classes, and classes nested within centres (NHS districts). School was initially considered as a clustering level, however, likelihood ratio tests comparing models with and without school-level showed that its inclusion did not significantly improve the model fit. Therefore, the final models included random intercepts at class and centre levels leaving out the school level. Residual intraclass correlation at class-level accounted for 9.5% of variance (class ICC 0.095). The original “centre” variable included nine NHS districts, however some centres had low sample size and therefore were merged based on similar geographic and environmental characteristics, finally resulting in four groups: Rome (35.4%), Torino3/Torino5/Torino (28.5%), Vercelli/Cuneo1/Cuneo2 (12.7%), and Alessandria/Novara (23.4%). Adjusted Odds Ratios (AORs) and 95% Confidence Interval (95% CI) were estimated as measures of association between the factors and the outcome.

To improve interpretability of factors, some variables were categorised into dichotomous or tertile-based indicators. Some categorical variables were re-coded in order to reduce the number of items included in the model, i.e., categories were merged. Sensitivity analyses using the original scored variables were conducted to assess robustness of the variables: the overall pattern, direction, and significance of associations remained consistent with the primary analyses.

Exploratory cross-sectional indirect association analyses were conducted to test the role of school-related attitudes and behavioural traits in the relationship between contextual factors and adolescent’s lifetime antisocial behaviour using the PROCESS macro for SPSS (22). In these models, school-related attitudes (school motivation and respect for teacher) and behavioural traits (impulsiveness and sensation-seeking) were considered as factors that may be more directly associated with antisocial behaviour as they may represent an individual response to broader contextual influences. Six multivariate models were run, one for each contextual factor, adjusting for the same confounders (gender, age, household employment status, family structure, languages in family, centre, alcohol drinking, cigarette smoking, gambling). The indirect effect and 95% Confidence Interval were estimated through bootstrapping (1000). Finally, two sensitivity analyses were conducted to validate and test the goodness of the factors included in the multilevel mixed-effect models. The first using the outcome defined as “three or more antisocial behaviours” vs. “two or less antisocial behaviours”, the second excluding the item “got into fight with someone” from the overall outcome measurement (Cronbach’s *α* = 0.593). The overall pattern, direction and significance of associations remained broadly consistent with the results of the original analyses. The effect sizes slightly varied depending on outcome severity; however, the same contextual (perceived parental permissiveness to use substances, perceived parental support, perceived class climate, perception of peer’s cigarette, alcohol and illicit drug use) and individual traits (school motivation, respect for teacher, impulsiveness, sensation seeking) remained significantly related to the outcomes. The only exception was school motivation as it was only marginally associated with three or more antisocial behaviours.

Statistical analyses were carried out using STATA software release 18.0 and SPSS software release 28 (23, 24).

## 3 Results

### 3.1 Descriptive statistics

The overall prevalence of lifetime antisocial behaviours was 61.2%. Analysing antisocial behaviours separately, the most frequent was have stolen something (34.6%), followed by got into fight with someone (33.4%), have seriously hurt someone (29.4%), have destroyed on purpose someone’s items (25.6%), have hit one of the teachers (7.9%), have threatened someone with a weapon to get something (4.6%), and 3.6% have sold stolen items. Except for have threatened someone with a weapon to get something, all the other antisocial behaviours were significantly higher among boys compared to girls (Figure 1).

**Figure 1:**
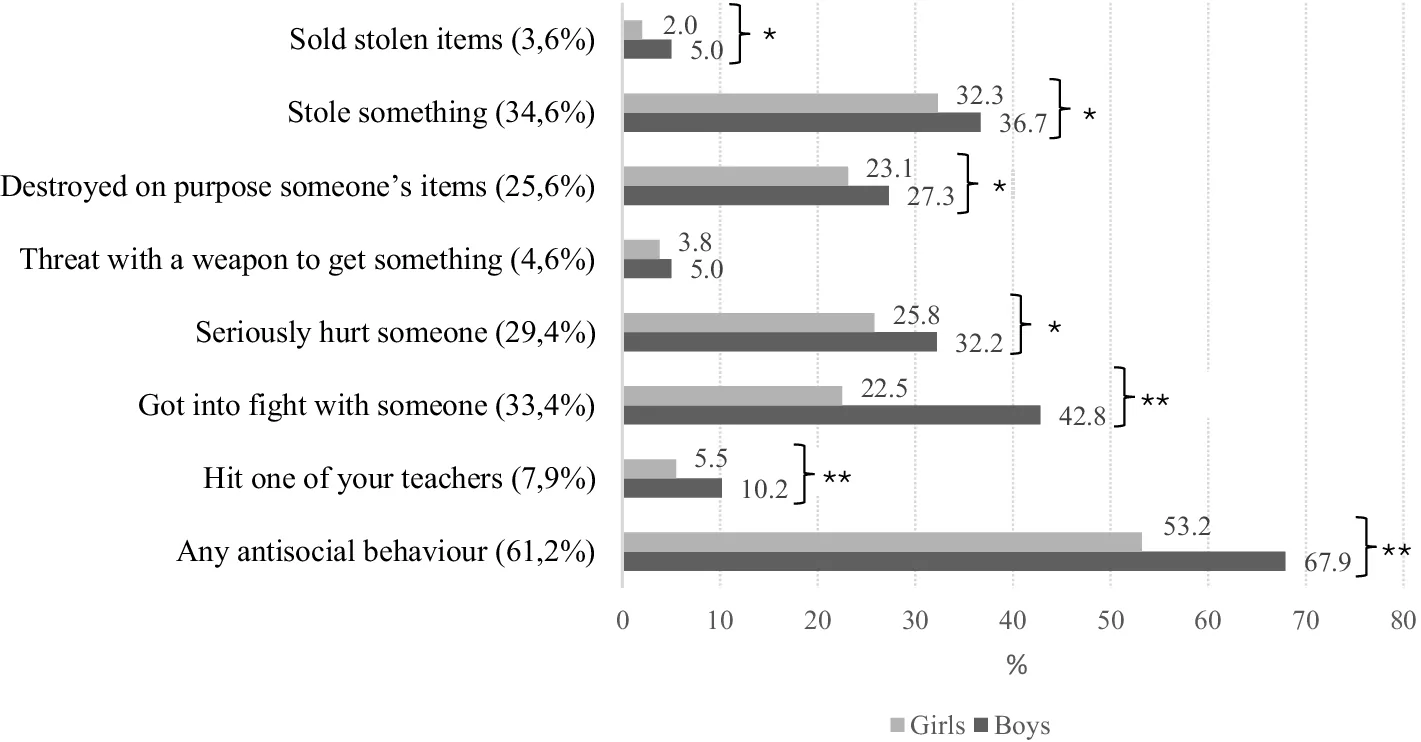
Lifetime prevalence of antisocial behaviours by gender *p-value <0.050, **p-value < 0.001.

Figure 2 shows the distribution of the number of antisocial behaviours as reported by adolescents. About 22% of adolescents reported one antisocial behaviour lifetime, 17.3% reported two behaviours, 11.0% reported three behaviours, 6.2% reported four, 3.3% reported five, 1.1% reported six, and 0.2% reported seven behaviours. Overall, outcomes estimated in sensitivity analysis accounted for 21.8% (adolescents reporting three or more antisocial behaviours) and 55.2% (overall outcome excluding “got into fight with someone”) of cases.

**Figure 2:**
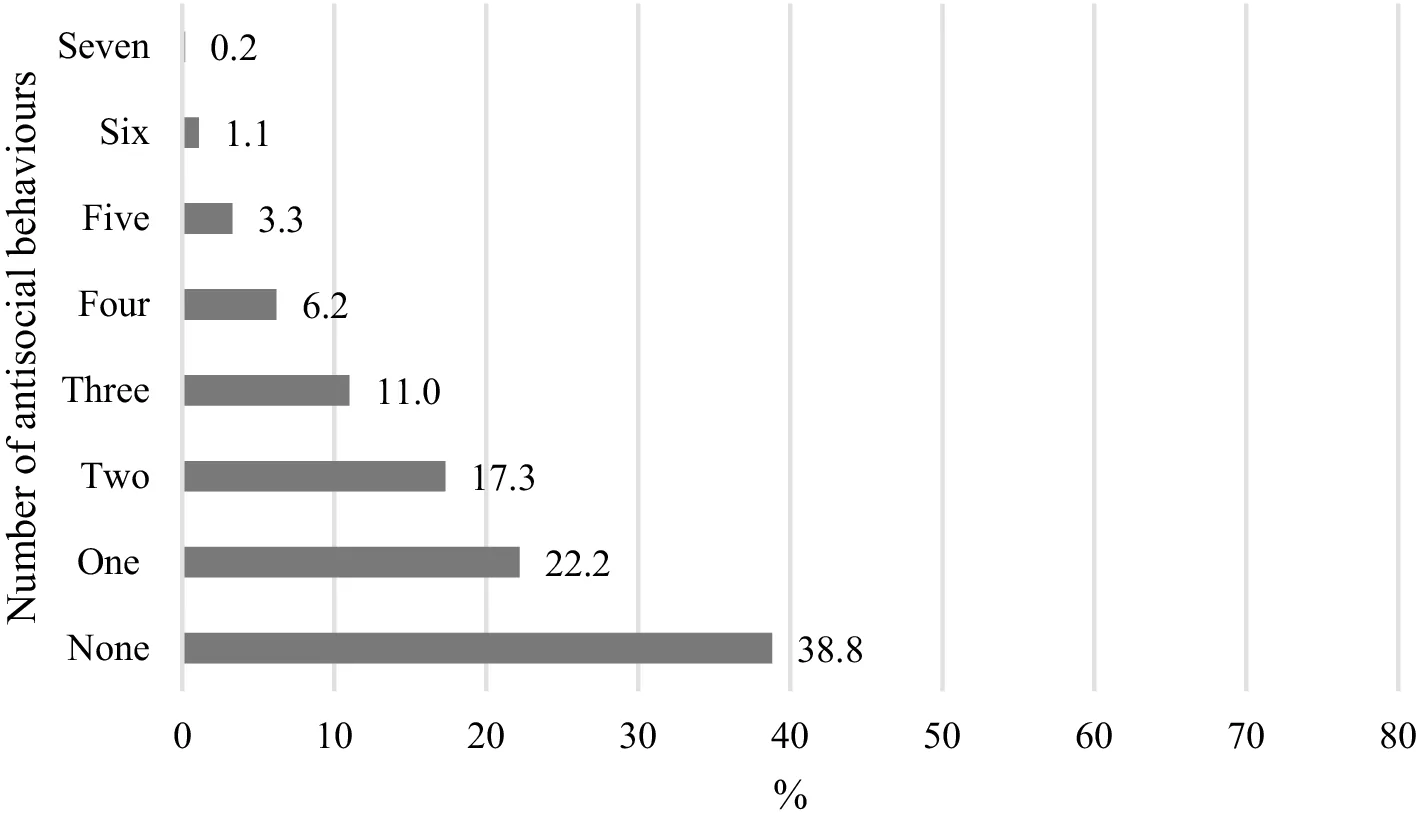
Lifetime prevalence of antisocial behaviours by frequency.

The bivariate associations of contextual factors, school-related attitudes and behavioural traits and co-occurring risk behaviours with antisocial behaviours lifetime are shown in Table 1. Boys were more prevalent in antisocial group compared to girls (61.2% vs. 53.2%, *p* < 0.001). Mean age of the participants was 13.0 ± 0.8 years with no difference between pupils engaging or not in antisocial behaviour lifetime. A greater proportion of students from non-Italian speaking families reported antisocial behaviour (27.4% vs. 23.0%, *p* = 0.036). Age, household employment status and family composition did not differ significantly between pupils engaging or not in lifetime antisocial behaviour.

**Table 1:** Socio-demographic characteristics, contextual factors, school-related attitudes, behavioural traits and co-occurring risk behaviours of pupils engaging or not in antisocial behaviour lifetime.

Characteristics	Overall (*N* = 1847)		Antisocial behaviour (N = 1,131)		No antisocial behaviour (*N* = 716)		*p*-value
	*N*	%	*N*	%	*N*	%	
Age							
12	524	28.4	315	27.9	209	29.2	0.720
13	826	44.7	505	44.7	321	44.8	
14	497	26.9	311	27.5	186	26.0	
Mean±SD	13.0 ± 0.8		13.1 ± 0.8		13.1 ± 0.8		0.243
Gender							
Girls	842	48.2	448	42.2	394	57.6	<0.001
Boys	904	51.8	614	57.8	290	42.4	
Languages in family							
Italian	1,368	74.3	818	72.6	550	77.0	0.036
Others	472	25.7	308	27.4	164	23.0	
Household employment status							
Two salaries	1,358	74.2	836	74.8	522	73.4	0.517
One or no salary	471	25.8	282	25.2	189	26.6	
Family composition							
Both parents	1,445	78.4	873	77.4	572	79.9	0.404
One parent	189	10.2	123	10.9	66	9.2	
Others	210	11.4	132	11.7	78	10.9	
Perceived parental permissiveness to use substances							
Would not allow at all	1,292	70.9	713	63.8	579	82.0	<0.001
Would not allow at home/Would allow	531	29.1	404	36.2	127	18.0	
Perceived parental support							
High	1,581	86.3	936	83.5	645	90.7	<0.001
Low	251	13.7	185	16.5	66	9.3	
Perceived class climate							
High	821	44.5	465	41.2	356	49.8	<0.001
Middle	531	28.8	318	28.2	213	29.8	
Low	491	26.6	345	30.6	146	20.4	
Perception of peer’s cigarette use							
No	302	16.6	148	13.3	154	21.8	<0.001
Yes	1,519	83.4	967	86.7	552	78.2	
Perception of peer’s alcohol use							
No	549	30.3	302	27.2	247	35.3	<0.001
Yes	1,261	69.7	808	72.8	453	64.7	
Perception of peer’s illicit drug use							
No	1,041	57.9	594	53.8	447	64.3	<0.001
Yes	758	42.1	510	46.2	248	35.7	
School motivation							
High	1,587	86.4	926	82.4	661	92.8	<0.001
Low	249	13.6	198	17.6	51	7.2	
Respect for teacher							
High	1,616	88.2	936	83.6	680	95.5	<0.001
Low	216	11.8	184	16.4	32	4.5	
Impulsiveness							
Low	767	41.7	325	28.8	442	62.3	<0.001
Middle	478	26.0	310	27.5	168	23.7	
High	593	32.3	493	43.7	100	14.1	
Sensation-seeking							
Low	756	41.1	321	28.5	435	61.0	<0.001
Middle	495	26.9	319	28.4	176	24.7	
High	587	31.9	485	43.1	102	14.3	
Cigarette smoking							
No	1,610	87.2	922	81.5	688	96.1	<0.001
Yes	237	12.8	209	18.5	28	3.9	
Alcohol drinking							
No	1,290	69.8	693	61.3	597	83.4	<0.001
Yes	557	30.2	438	38.7	119	16.6	
Gambling							
No	807	44.3	415	37.2	392	55.6	<0.001
Yes	1,015	55.7	702	62.8	313	44.4	

Standard Deviation (SD).

Cigarette smoking and alcohol drinking were measured in lifetime.

Gambling behaviour was measured in the last 12 months.

As regards contextual factors, perceived parental permissiveness towards substance use (36.2% vs. 18.0%, *p* < 0.001), low perceived parental support (16.5% vs. 9.3%, *p* < 0.001), and low perceived class climate (30.6% vs. 20.4%, *p* < 0.001) were more prevalent among students who reported antisocial behaviour. Pupils of the antisocial group had a significantly higher perception of peer’s cigarette use (86.7% vs. 78.2%, *p* < 0.001), alcohol use (72.8% vs. 64.7%, *p* < 0.001) and illicit drug use (46.2% vs. 35.7%, *p* < 0.001) compared to those in non-antisocial group.

In terms of school-related attitudes and behavioural traits, low school motivation (17.6% vs. 7.2%, *p* < 0.001) and low respect for teacher (16.4% vs. 4.5%, *p* < 0.001) were significantly higher among pupils who reported antisocial behaviour. High impulsiveness (43.7% vs. 14.1%, *p* < 0.001), and high sensation-seeking (43.1% vs. 14.3%, *p* < 0.001) were significantly higher among those in the antisocial group.

As regards co-occurring risk behaviours, a higher prevalence of lifetime cigarette smoking (18.5% vs. 3.9%, *p* < 0.001), lifetime alcohol drinking (38.7% vs. 16.6%, *p* < 0.001) and last 12 months gambling (62.8% vs. 44.4%, *p* < 0.001) was observed in the antisocial group.

### 3.2 Multivariate mixed-effect regression models

The association of contextual factors with lifetime antisocial behaviour is shown in Table 2. In Model 1, perception of parental permissiveness to use substances (AOR 1.79, 95% CI 1.34–2.39), low perceived parental support (AOR 1.74, 95% CI 1.21–2.50) and low perceived class climate (AOR 1.92, 95% CI 1.46–2.52) were significantly associated with the odds of adolescent’s lifetime antisocial behaviour. Perceptions of peer’s cigarette use (AOR 1.92, 95% CI 1.40–2.62), alcohol use (AOR 1.30, 95% CI 1.00–1.69) and illicit drug use (AOR 1.45, 95% CI 1.13–1.85) were also significantly associated with higher odds of involvement in antisocial behaviour. In Models 2 and 3, perception of parental permissiveness to use substances, low perceived parental support and low perceived class climate were associated with the odds of lifetime antisocial behaviour similarly as in Model 1.

**Table 2:** Contextual factors associated with lifetime antisocial behaviour.

Contextual factors	Model 1 (*N* = 1,645)		Model 2 (*N* = 1,635)		Model 3 (*N* = 1,623)	
	AOR (95% CI)	*p*-value	AOR (95% CI)	*p*-value	AOR (95% CI)	*p*-value
Perceived parental permissiveness to use substances						
Would not allow at all	1		1		1	
Would not allow at home/Would allow	1.79 (1.34–2.39)	<0.001	1.73 (1.30–2.31)	<0.001	1.77 (1.32–2.35)	<0.001
Perceived parental support						
High	1		1		1	
Low	1.74 (1.21–2.50)	0.003	1.69 (1.18–2.42)	0.004	1.64 (1.14–2.35)	0.007
Perceived class climate						
High/Middle	1		1		1	
Low	1.92 (1.46–2.52)	<0.001	1.90 (1.45–2.48)	<0.001	1.86 (1.42–2.44)	<0.001
Perception of peer’s cigarette use						
No	1					
Yes	1.92 (1.40–2.62)	<0.001	—		—	
Perception of peer’s alcohol use						
No			1			
Yes	—		1.30 (1.00–1.69)	0.046	—	
Perception of peer’s illicit drug use						
No					1	
Yes	—		—		1.45 (1.13–1.85)	0.003

Multilevel mixed-effect models controlled for two levels: centre and class.

The models were adjusted for gender, age, language in family, household employment status, family structure, alcohol drinking, cigarette smoking, gambling.

Adjusted Odds Ratios (AOR); 95% Confidence Interval (95%CI).

Model 1: Multivariate regression model including the variable “Perception of peer’s cigarette use”.

Model 2: Multivariate regression model including the variable “Perception of peer’s alcohol use”.

Model 3: Multivariate regression model including the variable “Perception of peer’s illicit drug use”.

The association of school-related attitudes and behavioural traits with lifetime antisocial behaviours is shown in Table 3. Low school motivation (AOR 1.84, 95% CI 1.21–2.80), low respect for teacher (AOR 2.23, 95% CI 1.35–3.68), middle (AOR 2.09, 95% CI 1.57–2.78) and high (AOR 4.02, 95% CI 2.93–5.51) impulsiveness, and middle (AOR 1.84, 95% CI 1.39–2.45) and high (AOR 3.51, 95% CI 2.56–4.80) sensation seeking level were significantly associated with an increased odds of engagement in antisocial behaviours.

**Table 3:** School-related attitudes and behavioural traits associated with lifetime antisocial behaviour (*N* = 1,667).

School-related attitudes and behavioural traits	AOR (95% CI)	*p*-value
School motivation		
High	1	
Low	1.84 (1.21–2.80)	0.005
Respect for teacher		
High	1	
Low	2.23 (1.35–3.68)	0.002
Impulsiveness		
Low	1	
Middle	2.09 (1.57–2.78)	<0.001
High	4.02 (2.93–5.51)	<0.001
Sensation-seeking		
Low	1	
Middle	1.84 (1.39–2.45)	<0.001
High	3.51 (2.56–4.80)	<0.001

Multilevel mixed-effect models controlled for two levels: centre and class.

The model was adjusted for gender, age, language in family, household employment status, family structure, alcohol drinking, cigarette smoking, gambling.

Adjusted Odds Ratios (AOR); 95% Confidence Interval (95% CI).

### 3.3 Exploratory indirect associations models

Results of exploratory indirect associations models are shown in Table 4. Impulsiveness was a significant indirect correlate of the relationship between perceived parental permissiveness to use substances, perceived parental support, perceived class climate and perception of peer’s cigarette use and antisocial behaviour. Sensation-seeking was a significant indirect correlate for all contextual factors except for low class climate. Low pupil’s respect for teacher was a significant indirect correlate of the association between family-related factors and class climate. School motivation was not significant indirect correlate in any model. Impulsiveness and sensation-seeking were the only indirect correlates of the relationship between perceived peer’s cigarette use and antisocial behaviour. Sensation-seeking was the only indirect correlate for perceived peer’s alcohol and illicit drug use.

**Table 4:** Indirect effect of school-related attitudes and behavioural traits on the relationship between contextual factors and lifetime antisocial behaviour.

Contextual factors	School-related attitudes and behavioural traits	Path a		Path b		Path a*b
		*β* (95% CI)	*p*-value	*β* (95% CI)	*p*-value	*β* (95% CI)
Perception of parental permissiveness to use substances direct effect: *β* = 0.353 (0.061, 0.646), *p* = 0.018	School motivation	0.110 (0.018, 0.202)	<0.001	n.s.		n.s.
	Respect for teacher	0.098 (0.016, 0.181)	<0.001	0.436 (0.246, 0.626)	<0.001	0.043 (0.008, 0.090)
	Impulsiveness	0.148 (0.080, 0.216)	<0.001	0.985 (0.745, 1.222)	<0.001	0.146 (0.080, 0.222)
	Sensation-seeking	0.139 (0.077, 0.200)	<0.001	0.996 (0.744, 1.247)	<0.001	0.138 (0.070, 0.221)
Low perceived parental support direct effect: *β* = 0.373 (0.002, 0.743), *p* < 0.049	School motivation	n.s.		n.s.		n.s.
	Respect for teacher	0.340 (0.241, 0.439)	<0.001	0.412 (0.220, 0.603)	<0.001	0.140 (0.070, 0.231)
	Impulsiveness	0.114 (0.032, 0.196)	0.007	0.971 (0.736, 1.207)	<0.001	0.111 (0.025, 0.211)
	Sensation-seeking	0.086 (0.010, 0.161)	0.026	1.005 (0.757, 1.253)	<0.001	0.086 (0.006, 0.173)
Low perceived class climate direct effect: *β* = 0.437 (0.225, 0.649), *p* < 0.001	School motivation	0.148 (0.084, 0.212)	<0.001	n.s.		n.s.
	Respect for teacher	0.307 (0.251, 0.363)	<0.001	0.351 (0.158, 0.545)	<0.001	0.108 (0.052, 0.180)
	Impulsiveness	0.132 (0.085, 0.179)	<0.001	0.959 (0.724, 1.195)	<0.001	0.127 (0.073, 0.191)
	Sensation-seeking	n.s		1.062 (0.811, 1.314)	<0.001	n.s.
Perception of peer’s cigarette use direct effect: *β* = 0.400 (0.086, 0.714), *p* = 0.013	School motivation	n.s.		n.s.		n.s.
	Respect for teacher	n.s.		0.453 (0.260, 0.647)	<0.001	n.s.
	Impulsiveness	0.117 (0.039, 0.96)	0.004	0.976 (0.740, 1.212)	<0.001	0.114 (0.034, 0.208)
	Sensation-seeking	0.155 (0.083, 0.226)	<0.001	0.987 (0.738, 1.237)	<0.001	0.153 (0.072, 0.251)
Perception of peer’s alcohol use direct effect: *β* = 0.149 (−0.119, 0.417), *p* = 0.276	School motivation	n.s.		n.s.		n.s.
	Respect for teacher	n.s.		0.451 (0.258, 0.644)	<0.001	n.s.
	Impulsiveness	n.s.		0.990 (0.754, 1.226)	<0.001	n.s.
	Sensation-seeking	0.113 (0.052, 0.173)	<0.001	1.007 (0.756, 1.257)	<0.001	0.113 (0.046, 0.192)
Perception of peer’s illicit drug use direct effect: *β* = 0.355 (0.103, 0.606), *p* = 0.006	School motivation	n.s.		n.s.		n.s.
	Respect for teacher	n.s.		0.455 (0.261, 0.649)	<0.001	n.s.
	Impulsiveness	n.s.		0.968 (0.731, 1.205)	<0.001	n.s.
	Sensation-seeking	0.075 (0.020, 0.130)	0.008	1.003 (0.751, 1.254)	<0.001	0.075 (0.017, 0.140)

Not significant (n.s.); 95% Confidence Interval (95% CI).

All multivariate mediation models were adjusted for age, gender, centre, languages in family, household employment status, family structure, alcohol drinking, cigarette smoking, gambling.

Path a: effect of the contextual factors on targeted indirect correlates.

Path b: effect of targeted indirect correlates on the outcome.

Path a*b: indirect association of potential mediators.

In almost all models, individual traits were partial indirect correlates of the association between contextual factors and antisocial behaviours, as the direct effect remained significant in all multivariate mediation model. The only exception was the model including perceptions of peer’s alcohol use as the direct effect did not reach statistical significance.

## 4 Discussion

The present study examined the association of contextual factors with antisocial behaviour in early adolescents, as well as the indirect effect of school-related attitudes and behavioural traits on the relationship between contextual factors and adolescent’s antisocial behaviour. Among contextual factors, perceived parental permissiveness to use substances, perceived parental support, perceived class climate and perception of peer’s cigarette, alcohol and illicit drug use were significantly associated with an increased odds of adolescent’s engagement in antisocial behaviours. Low pupil’s respect for teacher, high impulsiveness and high sensation-seeking attitude were significant indirect correlates of the associations of most contextual factors on antisocial behaviours. These findings identify exploratory cross-sectional indirect associations that may help generate hypotheses for future longitudinal research. Although causal relationships cannot be established, the identified factors may capture important targets for prevention interventions.

In the present study, 61.2% of participating students reported engaging in antisocial behaviours lifetime (54.2% reported violence and 34.8% stealing). The most prevalent forms of antisocial behaviours were “got into fight with someone,” “seriously hurt someone,” “destroyed on purpose someone’s items” and “stole something.” However, when considering adolescents reporting three or more antisocial behaviours the prevalence was 21.8%. Albeit considering the differences in timeframe of the measure, the prevalence of any lifetime antisocial behaviour observed in our sample of 12–14 year-old adolescents appears to be noteworthy high compared to 18.3% prevalence of violent behaviour in the past 12 months found in 15–16 year-old ESPAD students (3). However, when focusing only on the most severe cases, the prevalence was similar to that observed in the ESPAD survey. Evaluating antisocial behaviours in adolescents may be challenging because of differences in conceptualization and measures, leading to inconsistencies among studies. In the present case, the timeframe of the measures can only partially explain the observed difference. Furthermore, the data from the ESPAD study were collected before the COVID-19 (2019 wave of the ESPAD Italia cross-sectional survey), whereas our study was conducted immediately after the COVID-19 (2022/2023). It can be speculated that the variation in pre-COVID and post-COVID circumstances could have impacted the adolescent’s proneness to get involved in antisocial behaviours due to isolation and other risk factors related to pandemic. This alarmingly high prevalence requires immediate actions in identifying risk factors early in life and in implementing targeted and effective prevention programs prior the onset of antisocial behaviour. As expected, the prevalence of antisocial behaviours was higher among boys than girls, consistently with several previous studies (3, 11). Fagan et al. (11) suggested that boy’s greater involvement in antisocial behaviours is a result of the combination of their higher and significantly stronger exposure to risk factors, and lower exposure to protective factors compared to girls. Based on these findings, authors suggested that boys could experience greater benefits from participation in interventions for preventing antisocial behaviours (11), a hypothesis that we can support due to previous results on gender differences in effectiveness of the Unplugged program we observed in the EU-Dap study (25).

The perception of parental permissive attitudes towards substance use was associated with antisocial behaviours, as already observed elsewhere (4). The association was potentially mediated by low respect for teacher, consistently with previous studies that found significant effect of negative school factors on antisocial behaviours (4, 11, 12). High impulsiveness and sensation-seeking attitude also acted as indirect correlates of this association. These are internal states that can amplify the influence of external factors; indeed, adolescents who are highly impulsive or sensation-seekers may be more prone to act out antisocially (9, 11, 12, 16, 17), especially if contextual factors provide permissive messages. Furthermore, parents who approve the use of substances may send a message to their offspring that risk behaviours are acceptable, possibly lowering their repulsiveness against engaging in antisocial activities. Indeed, several previous studies found that parental permissiveness toward the use of one substance can be generalized to the use of other substances, and to other risk behaviours in general (4, 26–28).

In terms of parent–child relationship, low perceived parental support increased the odds of engaging in antisocial behaviours, consistent to prior studies that reported low parental emotional support, negative family climate and family conflicts as significant correlates of this behaviour (3, 4, 6, 7, 9, 10). Again, the association was potentially mediated by low respect for teacher, high impulsiveness and high sensation-seeking attitude. Coherently, Houltberg et al. (13) found that parental support was indirectly related to prosocial behaviour through anger regulation. These findings suggest that promoting the control of impulsiveness and anger are important aspects of prevention strategies aimed to reduce this risk behaviour among adolescents.

Pupils perceiving low class climate showed a more pronounced odds of engaging in antisocial behaviour. For this relationship, both school related factors, i.e., low respect for teacher, and personal factors, i.e., high impulsiveness (but not sensation seeking) were potential mediators. Low respect for teachers may be an important indicator of student’s engagement with structured school environment. Poor performance or disrespect can reflect underlying disconnection from social norms and proneness to antisocial behaviours, which may be exacerbated by poor class climate. Similarly, several previous studies reported that weak school bonding and low commitment to school increase the susceptibility to engage in antisocial behaviours (4, 9, 11).

Adolescents who perceived high prevalence of peer’s use of cigarettes, alcohol and illicit drugs were particularly prone to antisocial behaviours, similarly to several previous studies that observed the link of antisocial behaviours with peer’s drug use and attachment to delinquent peers (4, 9, 11, 12, 16). Peers have a strong influence in shaping social norms of adolescents favouring the perception of risk behaviours as acceptable. In these relationships, sensation-seeking acted as intermediate correlate between perception of peer’s use of all substances and antisocial behaviours, whether impulsiveness acted as indirect correlate only for perception of peer’s cigarette use. Also in this case, impulsiveness and sensation-seeking may exacerbate the influence of external factors, particularly if peer’s norms are perceived as acceptable.

Deeper understanding of the mechanisms by which contextual factors affect the individual behaviours is of extreme importance as problem behaviours generally initiate during early adolescence and the knowledge of relationships and trajectories is crucial for targeting appropriate prevention strategies before the initiation of the behaviours. Our study suggests potential mediators that warrant future longitudinal investigation through temporal mediation models. According to results of the present study, empowerment of protective factors such as parental disapproval toward substance use, strong parental support, high class climate and correction of normative beliefs should be targeted, and tested, in intervention studies aimed to prevent antisocial behaviour.

This study has several strengths. The survey used a standardised questionnaire containing previously validated questions derived from recognized international sources, minimizing possible misclassifications related to data collection and measures. Multilevel mixed-effect regression models were performed to evaluate the association between correlates and antisocial behaviour, according to higher order clustering (centre and class). The information collected through the questionnaires allowed the analysis of a large set of correlates. The sample was large.

However, the study should be considered also in light of some limitations. The cross-sectional design may prevent inferring causality. Timing between exposure, potential mediators and outcome cannot be established and reverse associations are possible. Therefore, the results should be interpreted with caution as do not represent the evidence of temporal mediation or causal pathways but rather a suggestion of indirect associations that warrant future longitudinal investigation. However, the findings give a contribution to the growing body of research suggesting that family, peers and school environment factors are inter-related in the pathways that influence the development of antisocial behaviours in adolescents. Missing values reduced the analysis sample in the regression model, however missing data were less than 6% for all studied variables, therefore models were still run on a large sample. This is a secondary analysis of the study, i.e., the study was designed for a different purpose, and this could limit its representativeness. All the information was self-reported by the students; therefore, the pupil’s perceptions of peer’s substance use, parental permissiveness to use substances, parental support and class climate may not accurately reflect the actual behaviour or the contextual reality but rather the subjective perception. Nevertheless, students’ perceptions may have a strong impact on their behaviour. The outcome was an overall measure of antisocial behaviours lifetime including various items, from stealing to violent behaviours, with a very high prevalence. However, the robustness of the main findings were confirmed by sensitivity analyses conducted on more severe outcomes. Residual confounding remains possible, as several potentially important correlates of antisocial behaviour were not available in the dataset and therefore could not be controlled for. Last, the sample was not equality distributed between the nine centres, therefore some centres needed to be merged in order to perform multilevel analysis, and clustering was not fully accounted for in the PROCESS-based mediation models.

In conclusion, the investigation of the role of contextual factors, school-related attitudes and behavioural traits helps to understand antisocial behaviours in adolescence. Individual traits are particularly important since they may explain the association of contextual factors with the behaviour. These findings may help to generate hypotheses for future longitudinal studies. All these factors should be considered in prevention interventions aimed at reducing or preventing antisocial behaviours among early adolescents. Timely identification of youth at high risk is of utmost importance to deliver targeted prevention strategies and prevent associated consequences.

## Data Availability

The raw data supporting the conclusions of this article will be made available by the authors, without undue reservation.

## Footnotes

1
https://eudap.eu

2
https://www.euda.europa.eu/

## Group member of the GAPUnplugged Coordination Group

The GAPUnplugged Coordination Group includes: Federica Vigna-Taglianti, Mariaelisa Renna, Chiara Sacchi, Giulia Giraudi (Department of Translational Medicine, University of Piemonte Orientale, Novara); Adalgisa Ceccano, Maria Ginechesi, Claudia Vullo, Pietro Casella (Department of Mental Health, Addiction Unit, ASL Roma1, Roma); Serena Vadrucci (Department of Prevention, Hygiene and Public Health Unit, ASL Città di Torino, Torino); Fabrizio Faggiano, Chiara Andrà, Emina Mehanović, Alberto Sciutto, Erica Viola, Marco Martorana, Matteo Pezzutto, Cristina Scalvini (Department of Sustainable Development and Ecological Transition, University of Piemonte Orientale, Vercelli); Gian Luca Cuomo, Laura Donati (Piedmont Centre for Drug Addiction Epidemiology, ASL TO3, Grugliasco, Torino).
